# Association Between Evening Light Exposure and Subjective Sleep Measures Among People Living With Dementia

**DOI:** 10.1093/geroni/igab046.1694

**Published:** 2021-12-17

**Authors:** Miranda McPhillips, Yeji Hwang, Sonia Talwar, Nancy Hodgson

**Affiliations:** 1 University of Pennsylvania, University of Pennsylvania, Pennsylvania, United States; 2 University of Pennsylvania, School of Nursing, Philadelphia, Pennsylvania, United States; 3 University of Pennsylvania, School of Nursing, philadelphia, Pennsylvania, United States

## Abstract

Excessive light exposure before bedtime can disrupt one’s circadian rhythm and can lead to poor sleep. The purpose of this study was to describe the relationship between evening light exposure and subjective sleep measures in people living with dementia (PLWD). We conducted secondary data analysis using the baseline data from Healthy Patterns Clinical Trial (N=137). We used Actiwatch Spectrum Plus to collect light data over three consecutive days. We defined evening light exposure as the average white light intensity for 4 hours before sleep. Sleep measures included Epworth Sleepiness Scale and PROMIS Sleep-Related Impairment. We used univariate regression analysis. We found that that greater evening intensity of light exposure was associated with higher daytime sleepiness (β=0.209, p=0.015) and more sleep impairment (β=0.228, p=0.014). The results of our study suggest that exposure to bright light during evening can disturb nighttime sleep and increase daytime sleepiness in PLWD.

